# A Swelling over Sternum in a Child: Reminder of an Uncommon Diagnosis

**DOI:** 10.1155/2016/3765786

**Published:** 2016-10-25

**Authors:** Pradnya Joshi, Sandeep B. Bavdekar, Sushma U. Save

**Affiliations:** Department of Pediatrics, TN Medical College and BYL Nair Charitable Hospital, Mumbai, India

## Abstract

Lack of awareness about isolated tubercular osteomyelitis of the sternum resulted in a delay in diagnosing the condition in an eleven-year-old girl who presented with a gradually increasing swelling over the sternum. Radiological, histological, and microbiological investigations helped diagnose the condition and the child responded well to antitubercular therapy (ATT) and surgical debridement. The report provides a brief description about the various management options available.

## 1. Introduction

With only a handful of childhood cases of tubercular osteomyelitis of the sternum described in the literature [1–3], many pediatricians have not had the opportunity to see and manage such cases. This communication describes a case afflicted with the condition with an aim of increasing awareness about tuberculosis of the sternum.

## 2. Case History

**Figure 1:**
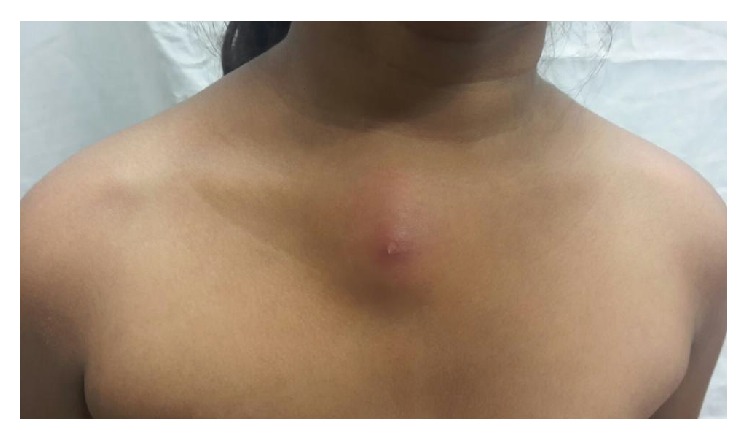
Clinical photograph showing swelling over the sternum.

An eleven-year-old girl was admitted with complaints of a gradually increasing circular nonpainful swelling (diameter 10 cm) over the sternum for the past 3 months (Figure 1). There was no history of local trauma or other symptoms such as fever, rash, or swellings over the body or bleeding from any site. There was no contact with tuberculosis. The patient had received oral analgesics, antipyretic agents, and antimicrobial agents during the intervening period. The patient's vital parameters were stable (pulse: 82/min, respiratory rate: 22/min, and BP: 108/64 mmHg) and anthropometric measurements were within centiles (weight: 30 kg, between 5th and 25th percentile; height: 129 cm, between 95th and 97th percentile; charts provided by the Centers for Disease Control, Atlanta). This child with BCG scar had no pallor, cyanosis, icterus, petechiae, ecchymoses, or lymphadenopathy. There were no swellings over the skull. The systemic examination was not contributory. The firm, nontender swelling with erythematous surface extended from manubrium to the sternal body. The child was investigated with the possible clinical diagnoses of tuberculous osteomyelitis and Langerhans cell histiocytosis.

**Figure 2:**
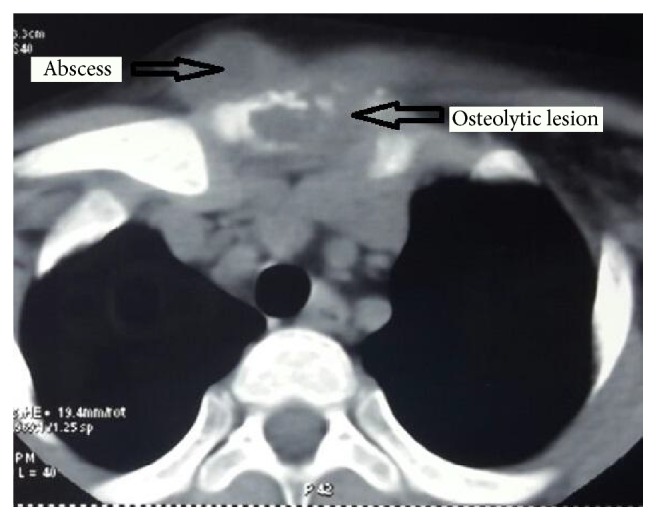
Computed tomography image demonstrating lytic destruction of manubrium with overlying peripherally enhancing soft tissue which is suggestive of osteomyelitis of the manubrium with abscess formation in the adjacent tissue.

The child's hemogram was normal (hemoglobin concentration: 10.4 gm/dL; total leukocyte count: 10800/mm^3^; platelet count: 347000/mm^3^; no abnormal cells on the peripheral smear). Raised erythrocyte sedimentation rate (50 mm at one hour) and positive Mantoux test (2 tuberculin units, induration: 16 mm) supported a diagnosis of tuberculosis, despite a normal chest radiograph and sputum smear negative for acid-fast bacilli (AFB). The test for human immunodeficiency virus (HIV) antibodies was negative. Fine needle aspiration cytology (FNAC) showed a cellular smear with intact and degenerated polymorphs, lymphocytes, and macrophages on caseous necrotic background suggestive of a cold abscess. The CT scan of the chest and thorax showed a lytic lesion of the manubrium sternum with an overlying soft tissue (Figure 2) without any evidence of pleural effusion, enlarged mediastinal lymph nodes, or parenchymal lesions. The patient was started on four-drug intensive antitubercular therapy (ATT) consisting of isoniazid, rifampicin, pyarzinamide, and ethambutol in appropriate doses. The lesion was subjected to debridement and curettage. The debrided material demonstrated fragments of fibrocollagenous tissue bits with granualation tissue and foci of caseous necrosis with the presence of epithelioid cells and granulomas with Langerhans type of giant cells without any malignant cells on microscopic examination. The material obtained at debridement grew* Mycobacterium tuberculosis* on culture with the organism being susceptible to the first-line antitubercular drugs. The FNAC and biopsy tissue sent for cartridge-based nucleic acid amplification technique (CB-NATT) were returned as the tissues had undergone degeneration. The ATT was continued and the child demonstrated complete resolution and healing of the lesion at follow-up visit 2 months later. At the last follow-up visit six months into the treatment, it was noted that she remained asymptomatic and had gained 5 kg. It is planned to continue ATT for a total period of 12 months.

## 3. Discussion

Isolated tubercular osteomyelitis is quite rare in children [1–3] and we suspect that consequent nonfamiliarity with the condition was responsible for the diagnosis getting delayed by over three months. It can occur through reactivation of latent foci formed during the initial hematogenous or lymphatic dissemination of tubercular organisms or by extension from adjacent and contiguous mediastinal lymph nodes [4]. The delay in the diagnosis can be hazardous as it has the potential to cause serious complications such as secondary infection, fistula formation, spontaneous sternum fracture, compression or erosion of large blood vessels, tracheal compression, or extension of sternal abscess into the mediastinum, subcutaneous tissue, or pleural cavity and infiltration into the bone marrow [4].

Tubercular osteomyelitis of the sternum presents with local symptoms like swelling [1, 2, 4–7], discharge [2, 7, 8], or ulcer [4] with [4, 6, 8, 9] or without [3] associated pain. Systemic manifestations such as cough [5, 7], fever [1, 2, 5, 8], night sweats [6–8], weight loss [2, 5–8], malaise [6], and fatigue [6], even when reported, are generally less dramatic. Injury to the local site has been reported as a trigger in a few cases [7, 10].

The diagnosis of the condition is generally delayed by months [1, 2, 4–6, 8, 9]. Besides, the paucity of experience with the condition among doctors, the nonspecific nature of initial symptoms, paucibacillary nature of the disease, analgesic and anti-inflammatory drugs providing partial or short-lasting relief of main symptoms, and antimicrobial agents providing some relief in the presence of a secondary bacterial infection contribute to this delay. Chest radiographs (especially the anteroposterior views) may not show bone lysis, sclerosis, periostitis, osteoporosis, osteopenia, or sternal fracture [9] in the initial stages and these normal radiographs may pose diagnostic problems for the practitioners. Most physicians consider pyogenic infections, malignancy (including lymphoma or metastatic cancers), granulomatous lesions (sarcoidosis, actinomycosis, and fungal infections), and Brodie's abscess in the differential diagnosis [9]. Ultrasonography can demonstrate the presence of soft tissue enlargement along with sinus formation [4]; the extent of bone and joint destruction [9] can be seen on the CT scan, even when the chest radiograph is normal. The CT scan can help detect mediastinal lymph nodes, which could be the underlying cause for sternal involvement. The HRCT may additionally help detect a parenchymal lesion not visualized on the chest radiograph. The MRI scan demonstrates abscess formation, bone marrow invasion [4], and the extent of the soft tissue mass even better than the CT scan. However, these imaging modalities and radionuclide scan can only define the extent of involvement and demonstrate the presence of complications. Only histopathological and microbiological investigations can help identify the etiology.

Antitubercular therapy is the mainstay of treatment and may be the only therapeutic modality needed to treat the condition [1, 2, 4]. Generally, the duration of ATT is 9 months; however, it can last for a longer period in children with extrapulmonary disease at the discretion of the treating physician [11]. We decided to prescribe ATT for 12 months in view of the sternum being an unusual extrapulmonary site. Nonresponse to medical treatment (persistence of discharge, nonhealing of ulcer, or persistence of constitutional symptoms) or presence of a large ulcer obviously mandates surgical intervention. As stated earlier, the need for histological or microbiological confirmation of diagnosis might necessitate debridement or aspiration. A school of thought proposes that such surgical intervention in all cases might promote healing, avert formation of a draining sinus, and prevent recurrence [3].

The diagnosis of isolated tubercular osteomyelitis of the sternum in children is a rarity even in India, where tuberculosis is endemic, resulting in delayed diagnosis. Fortunately, the child did not suffer from any complications and responded well to the first-line ATT. This case report with a brief overview of management modalities will serve as a reminder to doctors about this diagnosis and help minimize the delay in diagnosis and management. It should also be mentioned that judicious use of appropriate newer diagnostic techniques such as cartridge-based nucleic acid amplification techniques, heminested, cartridge-based real time PCR, and line probe assays can also help expedite the diagnostic process and provide clues regarding presence of a drug-resistant infection. Hopefully, these will ensure that children would be spared of unwarranted antibacterial therapy, repeated aspirations, and possibility of extension or complications of the disease.
